# The anti-apoptotic form of tyrosine kinase Lyn that is generated by proteolysis is degraded by the N-end rule pathway

**DOI:** 10.18632/oncotarget.1931

**Published:** 2014-05-01

**Authors:** Mohamed A. Eldeeb, Richard P. Fahlman

**Affiliations:** ^1^ Department of Biochemistry, University of Alberta, Edmonton Alberta Canada; ^2^ Department of Chemistry, Faculty of Science, Cairo University, Cairo, Egypt; ^3^ Department of Oncology, University of Alberta, Edmonton Alberta Canada

**Keywords:** Lyn, N-end rule, ubiquitination, protein degradation, UBR

## Abstract

The activation of apoptotic pathways results in the caspase cleavage of the Lyn tyrosine kinase to generate the N-terminal truncated LynΔN. This LynΔN fragment has been demonstrated to exert negative feedback on imatinib induced apoptosis in chronic myelogenous leukemia (CML) K562 cells. Our investigations focus on LynΔN stability and how reduced stability reduces imatinib resistance. As the proteolytical generated LynΔN has a leucine as an N-terminal amino acid, we hypothesized that LynΔN would be degraded by the N-end rule pathway. We demonstrated that LynΔN is unstable and that its stability is dependent on the identity of its N-terminus. Additionally we established that LynΔN degradation could be inhibited by either inhibiting the proteasome or knocking down the UBR1 and UBR2 ubiquitin E3 ligases. Importantly, we also demonstrate that LynΔN degradation by the N-end rule counters the imatinib resistance of K562 cells provided by LynΔN expression. Together our data suggest a possible mechanism for the N-end rule pathway having a link to imatinib resistance in CML. With LynΔN being an N-end rule substrate, it provides the first example that this pathway can also provide a pro-apoptotic function as previous reports have currently only demonstrated anti-apoptotic roles for the N-end rule pathway.

## INTRODUCTION

Lyn is a member of the Src family of tyrosine kinases (SFKs) that is a pivotal signalling intermediary for signal transduction pathways involved in a broad range of cellular functions such as proliferation[1, 2], differentiation[3], cell migration[4], autophagy[5] and apoptosis[6]. The structure of the Lyn protein includes: (i) a unique N-terminal domain (SH4) that contains a myristoylation site and a palmitoylation site[7] required for membrane attachment, followed by (ii) two protein-protein interaction domains initially characterized in SFKs (SRC homology 2 and 3 domains, SH2 and SH3 respectively), and (iii) a kinase domain containing its catalytic activity.

The expression of Lyn has also been linked to cancer progression and drug resistance. While the development of imatinib (Gleevac), a tyrosine kinase inhibitor targeting the Bcr-Abl fusion protein for the treatment of chronic myeloid leukemia (CML), has greatly improved the control of CML a significant number of patient eventually develop imatinib resistance[8]. While the most common resistance mechanism is the sporadic mutations to Bcr-Abl other mechanisms occurring at significant frequency include the overexpression of Src family kinases like Lyn[9, 10].

**Figure 1:**
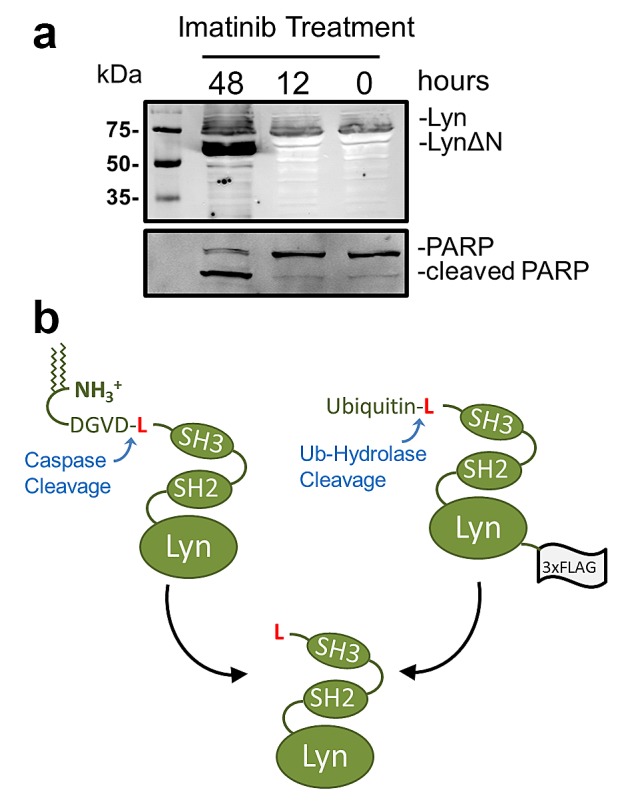
Protease dependent generation of LynΔN (a) K562 cells were transfected with a Lyn-GFP expressing vector. 24 hours after transfection, the cells were treated with 1μM imatinib for the indicated times. The cells were then lysed and resolved by SDS-PAGE for Western blot (WB) analysis. Western blot analysis reveals a faster migrating species after 48 hours of imatinib treatment. For WB analysis the polyclonal rabbit primary antibody used was either anti-GFP or anti-PARP (b) Schematic depiction of the generation of LynΔN by either caspase cleavage after Asp18 or as a recombinant ubiquitin fusion. Both methods of generating LynΔN in cells results in an N-terminal leucine.

During apoptosis in B cells, T cells and the CML cell line K562, Lyn is cleaved in its N-terminal SH4 domain by caspase-3 after aspartate 18, exposing a leucine as the new N-termini[11-13]. This cleavage shown schematically in Figure 1, was demonstrated to result in almost full length Lyn bearing an N-terminal leucine, but lacking the short N-terminal region containing the acylation sites necessary to maintain the association of Lyn to the plasma membrane. As a result, Lyn is free to diffuse from the plasma membrane into the cytosol of the cells [13]. The caspase-cleaved form of Lyn (LynΔN) was shown to exert an anti-apoptotic function in B-cells and K562 cells[11, 12]. In the case of the K562 cells, it was also demonstrated that LynΔN provided a significantly increased resistance in comparison to the full length un-cleaved Lyn protein[11]. Additional investigation in mouse models have revealed that the expression of LynΔN results in a psoriasis-like inflammatory syndrome and impair TFNR1 signalling[14, 15].

The N-end rule relates the *in vivo* half-life of a protein to the identity of its N-terminal amino acid residue[16, 17]. The components of the N-end rule pathway recognize proteins with specific N-termini and target these proteins for ubiquitin dependent degradation by 26 S proteasome. Similar but distinct versions of the N-end rule are present in all organisms from mammals to bacteria. In eukaryotes, N-end rule pathway mediated protein degradation has been implicated in diverse biological processes such as: G-protein signalling[18], DNA repair[19], cardiovascular development[20] and apoptosis[21].

The activity of the N-end rule pathway has been linked to the regulation of programmed cell death via the targeted degradation of proteolytic products that promote or carry out apoptosis [21-24]. In *D. melanogaster* it was demonstrated that the *Drosophila* inhibitor of apoptosis protein (DIAP1), which binds and inhibits active caspases, is cleaved by an active caspase. Caspase cleavage exposes an N-terminal asparagine which is an N-end rule destabilizing residue[21]. As a result, the N-end rule degradation machinery targets DIAP1, and potentially the active caspase, for degradation via the proteasome. More recent investigations with mammalian cells have shown that the N-end rule pathway targets a variety of pro-apoptotic protein fragments, generated as a result of proteolysis by active proteases during apoptosis, for degradation[23-25]. Furthermore, it was demonstrated that the partial ablation of the N-end rule pathway sensitizes mouse embryonic fibroblasts to apoptosis-inducing agents. Together, these results suggest a significant role for the N-end rule pathway on the suppression of the apoptotic program.

Here we investigate the role of N-end rule-mediated degradation of LynΔN, a caspase generated protein that counters cell death[11, 12]. We present the first study on the stability of LynΔN and demonstrate that the N-terminal leucine of LynΔN targets it for degradation via the N-End rule pathway. With the exception of pathogen induced cell death[26], this is the first example of the N-End rule functioning in a pro-apoptotic role by the targeted degradation of an anti-apoptotic protein.

## RESULTS AND DISCUSION

The proteolytic cleavage of Lyn by caspase-3 after aspartate 18 sheds the N-terminal segment of the protein that contains both the sites of N-myristoylation and S-palmitoylation[13]. The proteolytic release of the Lyn fragment has also been demonstrated to counter the apoptotic program and significantly increases the resistance of Ramos cells to IgM stimulation[12] and K562 cells to imatinib[11]. Figure 1a shows the Western blot analysis after expressing a Lyn-GFP construct in K562 cells, a CML derived cell line, treated with imatinib. Imatinib treatments results in the appearance of a faster migrating species that corresponds to the cleaved Lyn protein. Cleavage of Lyn is concomitant with PARP cleavage, which is commonly utilized to detect caspase activity[27]. The increased apparent amount of LynΔN over that of the full-length Lyn observed in the blot is attributed to poorer extraction of the full-length myristoylated and palmitoylated Lyn protein from the membrane insoluble fraction of the lysis buffer. The loss of the N-terminal domain of LynΔN with the fatty acid modifications results in the diffusion of LynΔN throughout the cytoplasm[12]. To date there has been no investigations into the stability of LynΔN.

We utilized an expression system to investigate the stability and cellular effects of LynΔN that is independent of caspase cleavage. This was to circumvent the challenges of having ongoing formation of the cleaved protein during investigations of protein stability, as the continued cleavage by caspase-3 would convolute the analysis. To mimic the caspase-cleaved form of the Lyn kinase, a plasmid construct was created to express a recombinant ubiquitin-LynΔN fusion protein with a C-terminal 3× FLAG tag as we have previously described[24]. The ubiquitin-fusion technique enables the expression of a protein with any desired N-terminal amino acid as the N-terminal ubiquitin is proteolytically removed by endogenous ubiquitin hydrolases, which releases LynΔN as generated by caspase-3 cleavage of the full length Lyn[13] (Figure 1b). This approach enables the expression of LynΔN with leucine as the N-terminal amino acid.

**Figure 2:**
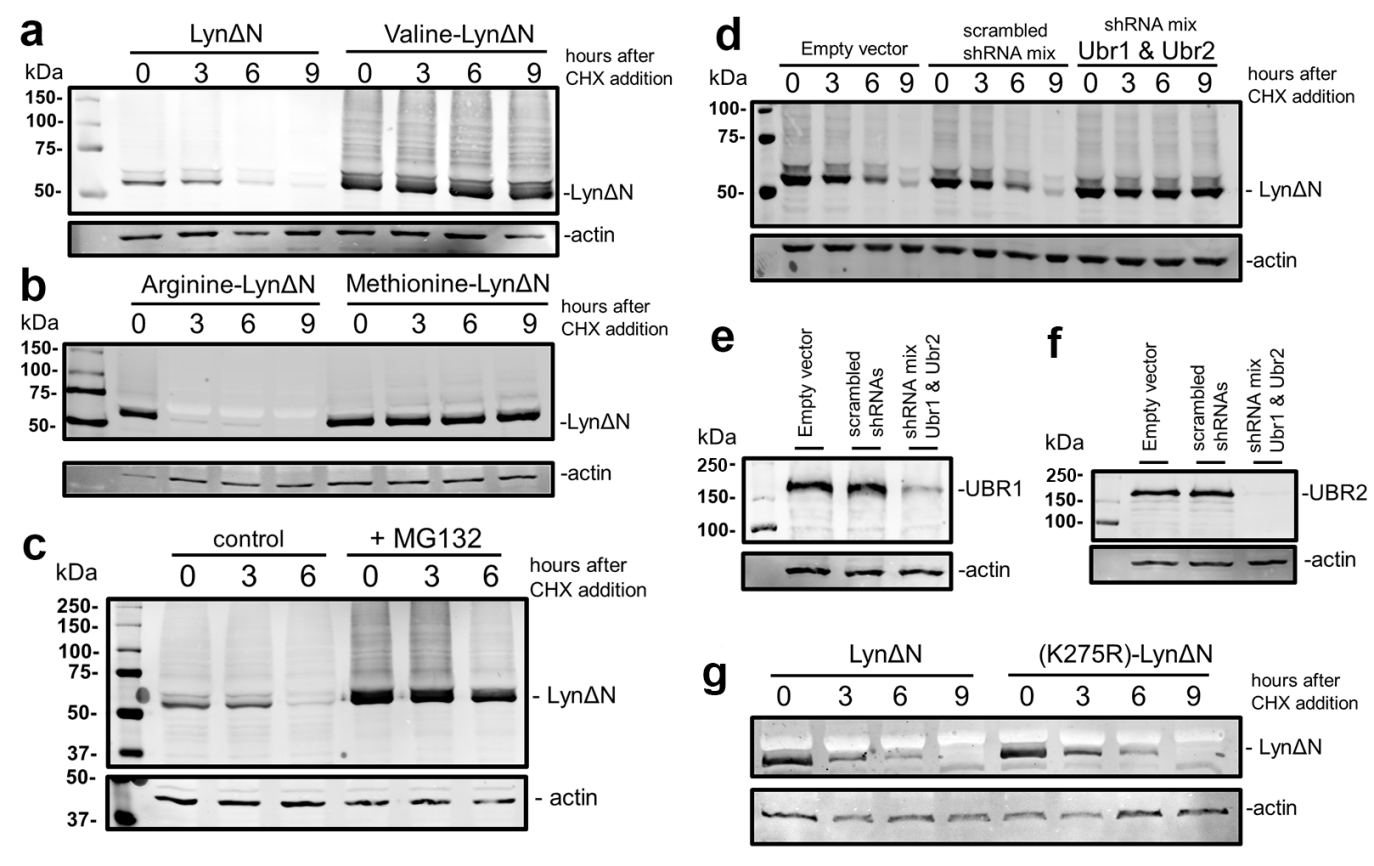
LynΔN is degraded by the N-end rule pathway (a) Wild type LynΔN and valine-LynΔN were transfected into HEK293T. After transfection the cells were treated with 100 μg/ml cycloheximide (CHX) to block protein synthesis. At the indicated time points, cells were lysed and resolved by SDS-PAGE. The amount of LynΔN protein remaining was visualized by WB analysis with an anti-FLAG M2 antibody Loading control analysis was done by WB analysis using an anti-actin antibody. (b) LynΔN with N-terminal arginine (type I destabilizing N-termini) and methionine (stable N-termini) were transfected into HEK293T and analyzed as in (a). (c) Stability of LynΔN was visualized in HEK293T cells in the presence and absence of 10 μM MG132 and analyzed as in (a). (d) Stability of LynΔN was visualized in HEK293T cells that were also transfected with a plasmid mixture expressing shRNAs targeting UBR1 and UBR2 or controls. Both vector control and plasmids expressing scrambled shRNA sequences were used as controls. In all cases the total amount of plasmid DNA was used for each transfection was kept constant. (e) Knock down of UBR1 after shRNA treatment in (d) was verified by WB analysis for endogenous UBR1 after SDS-PAGE on a 5% gel. (f) Knock down of UBR2 after shRNA treatment in (d) was verified by WB analysis for endogenous UBR2 after SDS-PAGE on a 5% gel. (g) Stability of LynΔN was compared to the stability of the inactive K275R-LynΔN mutant.

The N-terminal leucine of LynΔN renders it a potential N-End Rule substrate, as leucine is a type II primary destabilizing N-terminus[16]. To investigate this possibility we evaluated whether LynΔN was degraded and whether degradation was dependent on the identity of the N-termini. A mutation to the ubiquitin-LynΔN fusion was made to change the N-terminal leucine of Lyn to valine, methionine and arginine, as valine and methionine are stabilizing N-termini and arginine is a type I destabilizing N-terminus[16]. To investigate protein stability, the LynΔN constructs were transiently transfected into HEK293T. Twenty four hours following transfection, cells were treated with 100 μg/ml of cycloheximide (CHX) to inhibit protein synthesis and the cells were then lysed at increasing time points after CHX addition. Lysates were then resolved by SDS-PAGE and the amount of LynΔN remaining was quantified by Western blot analysis with an anti-FLAG antibody using actin as a loading control. The data reveals that LynΔN with the wild type leucine N-termini is unstable (Figure 2a). In contrast, the N-terminal valine mutant (Figure 2a) and methionine mutant (Figure 2b) are stable. As predicted the mutant with the N-terminal arginine is rapidly degraded (Figure 2b), as protein is only observed at the initial time point. This N-terminal dependent degradation of Lyn suggests that LynΔN may be a *bona fide* N-end rule substrate.

As the N-end rule pathway is an upstream branch of the ubiquitin proteasome-system, we then investigated whether the degradation of LynΔN is proteasome-dependent. We studied the degradation of the wild type LynΔN using the CHX protocol described above using HEK293T cells in the presence or absence 10 μM of MG132 proteasome inhibitor. The data shown in Figure 2c reveals that the addition of MG132 results in stabilization of LynΔN, demonstrating a role for the proteasome for LynΔN degradation.

The targeted degradation of proteins via the N-end rule pathway includes the recognition of the destabilizing N-termini by specific E3 ubiquitin ligases. To identify the E3 ubiquitin ligases that mediate the degradation of LynΔN, we initially investigated the role of E3 ubiquitin ligases UBR1 and UBR2. UBR1 and UBR2 are redundant E3 ubiquitin ligase previously identified to be components of the N-end rule pathway which when deleted in combination result in embryonic lethality[28]. To investigate whether UBR1 and UBR2 are required for degradation of LynΔN, the stability of LynΔN was investigated in co-transfection experiments with mixtures of plasmid vectors expressing shRNAs targeting UBR1 and UBR2. Both vector controls and plasmids expressing scrambled shRNA sequences were used as controls. In all cases the total amount of plasmid DNA was used for each transfection was kept constant. The data in Figure 2d reveals that LynΔN is unstable in both control experiments (scrambled shRNAs and vector control). In contrast, when UBR1 and UBR2 targeting shRNAs are simultaneously expressed the degradation of LynΔN is nearly completely inhibited. The knock down of both UBR1 and UBR2 by shRNA expression were verified by Western blot analysis for endogenous UBR1 (Figure 2e) and UBR2 (Figure 2f). The dependence on UBR1 and UBR2 for LynΔN degradation is in agreement with the hypothesis that degradation is via the N-end rule pathway.

As Lyn is a kinase, we investigate whether its activity is required for degradation via the N-end rule. An inactivating mutant (K275R), as previously described[29], was introduced into the wild type ubiquitin-LynΔN fusion construct. The stability of the wild type and K275R mutant LynΔN were then investigated as described above. As seen in Figure 2g, no significant change to the stability of the protein was observed when the enzymatic activity of LynΔN was blocked.

**Figure 3:**
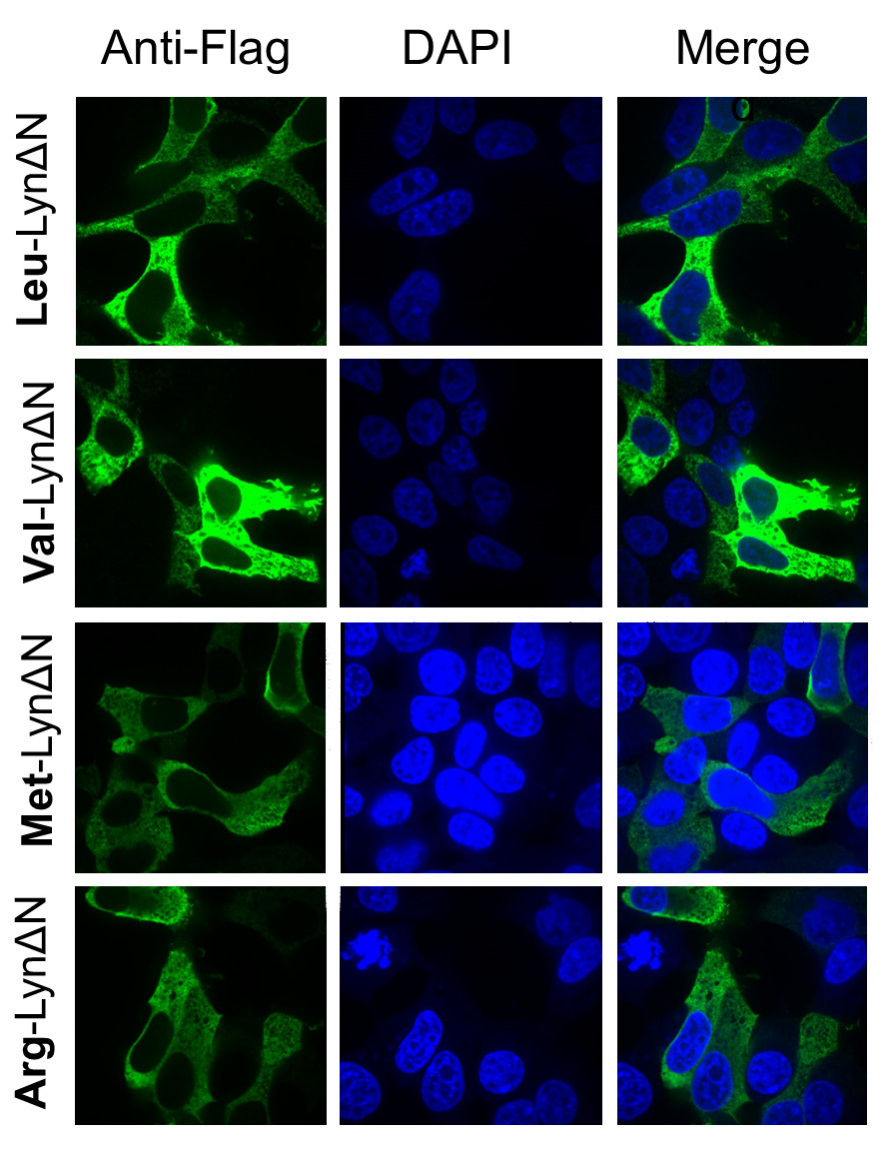
LynΔN is localized to the cytoplasm Immunofluorescent investigations of the cleaved fragment of Lyn (green) in transfected HEK293T cells in conjunction to nuclear staining with DAPI (blue). Localization of wild type LynΔN fragment N-terminal leucine (top) reveals a diffuse cytoplasmic localization. Localization of the mutant LynΔN fragments with the N-terminal valine, methionine and arginine all exhibit a similar cytoplasmic localization.

Initial investigations on LynΔN reported that this fragment localizes to the cellular cytoplasm versus the plasma membrane for the intact Lyn kinase[13]. We evaluated the localization of the recombinant LynΔN in HEK 293T cells to confirm its predicted cytoplasmic localization and that this localization is independent of the identity of the N-termini. Our results mimic the previously reported localization as the C-terminal fragment is localized to the cytoplasm (Figure 3). The cytoplasmic localization was independent of the identity of the N termini as identical results were obtained with fragments with N-terminal arginine, valine or methionin (Figure 3).

**Figure 4:**
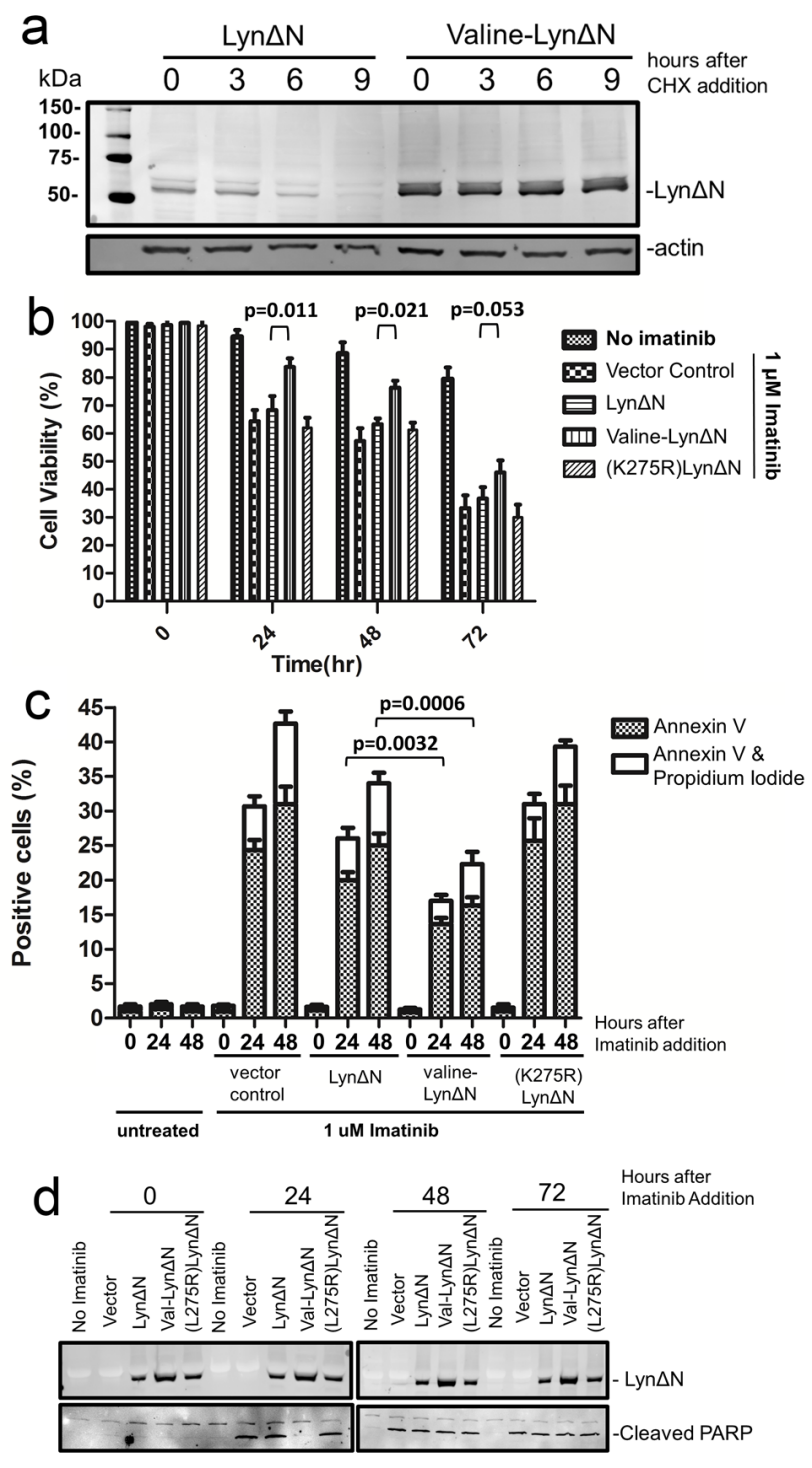
LynΔN induced imatinib resistance (a) Wild type LynΔN and valine-LynΔN were transfected into K562 cells, a CML derived cell line. After transfection the cells, CHX-chase experiment was done as previously described. The amount of LynΔN protein remaining was visualized by WB analysis with an anti-FLAG antibody and actin was analyzed as a loading control. (b) K562 cells were transfected to express the indicated proteins. Cells were then treated with imatinib for the indicated times then stained with trypan blue and analyzed on a TC20 automated cell counter (BioRad). The data represents the average and standard deviation from three independent experiments and p-values are derived from paired two tailed t-tests. (c) Quantified data from FACS analysis for K562 cells treated identically to those in (b). The percentage of cells that were stained with Annexin V (apoptotic cells) or both Annexin V and propidium iodide (dead cells) are shown. Cell staining was done with an Annexin V-FITC and propidium iodide. The data represents the average and standard deviation from three independent experiments. (d) Western Blot analysis of cell lysates from one set K562 cells used above. Blotting with an anti-FLAG antibody was used to detect LynΔN expression and an anti-PARP antibody was used to detect PARP cleavage.

We then investigated how the N-end rule degradation of LynΔN influences the previously reported anti-apoptotic role of this protein fragment[11]. Prior to investigating the cellular effects of LynΔN in K562 cells we first investigated that the LynΔN fragment was unstable in these cells as we observed for the HEK293T cells. The wild type LynΔN and the valine N-terminal mutant were both transfected into K562 cells and the stability of the proteins were investigated as described above with the utilization of cycloheximide. As observed in Figure 4a, the LynΔN with the wild type leucine N-termini was unstable while the valine N-terminal mutant was not degraded. We next investigated the sensitivity of K562 cells to imatinib that have been transfected with plasmids expressing either the wild type LynΔN, the stable valine N-terminal LynΔN mutant or a vector control. After transfection, 1 μM imatinib was added and the cells were evaluated by trypan blue exclusion staining to quantify cell viability after increasing times after imatinib addition. The data shown in Figure 4b reveal that the active degradation of LynΔN by the N-end rule significantly counters the imatinib resistance provided by LynΔN, this is demonstrated by the increased cell viability of K562 cells expressing the stable valine LynΔN mutant.

To verify that the increased viability during imatinib treatment as a result of LynΔN expression was a result of reduced apoptosis, we then proceed to analyze the cells for markers of apoptosis. The presence of phosphatidylserine on the outer leaflet of the plasma membrane[30] and caspase activity[27]. The cells were stained with Annexin V and propidium iodide (PI) and then analyzed by FACS. Data from analyses are summarized in Figure 4c, where the percentage of cells that were Annexin V positive or both Annexin V and propidium iodide positive is plotted. In agreement with the trypan blue staining, the expression of the valine-LynΔN resulted in the fewest number of apoptotic cells as observed by Annexin V and PI staining upon treatment of the cells with imatinib. FACS analysis was able to quantify a difference between the wild type LynΔN and the vector control, indicating that even with N-end rule degradation of LynΔN it can still function to counter the apoptotic stimuli to some extent. When the catalytically inactive form of LynΔN(K275R) is expressed, no significant change in the number of apoptotic cells was observed in comparison to the vector control. Western blot analysis to evaluate caspase activity by detecting caspase dependent PARP cleavage[27] is shown in Figure 4d. This data reveals that the expression of the stable valine-LynΔN protein results in a significant delay in the detection of the cleaved PARP fragment and thus caspase activation, where cleavage is only observed after 48 hours after the addition of imatinib. As a control for all the viability analysis we had also quantified the amount of expressed LynΔN in the cells (Figure 4d). As shown in the Western blot, all three forms were expressed but valine-LynΔN was detected in higher abundance. This increased amount is predicted as this is the stable form of LynΔN.

**Figure 5:**
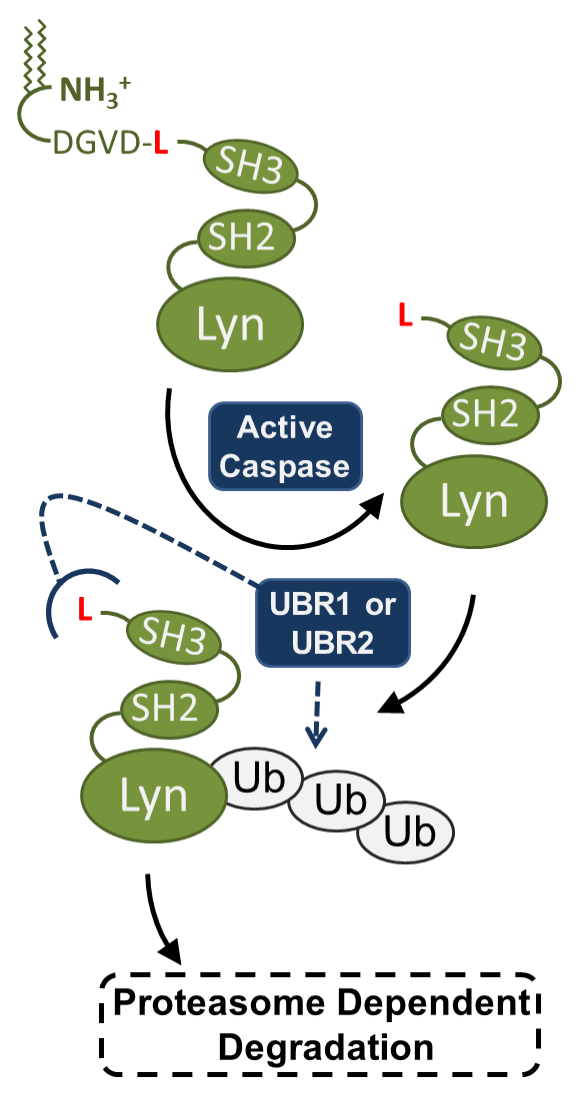
Model for LynΔN degradation Our final model is that the caspase generated LynΔN is recognized by the degenerate UBR1 and UBR2 E3 ubiquitin ligases. UBR1/2 recognize the N-terminal leucine of LynΔN which then ubiquitinates the protein, targeting it to the proteasome for degradation.

Together our data demonstrate that LynΔN is degraded in cells and this degradation is a result of the proteolytically exposed N-terminal leucine being recognized by the N-end rule machinery. In agreement with our proposed model of N-end rule degradation of LynΔN (Figure 5), we have demonstrated that degradation can be prevented by changing the identity of the N-terminal amino acid to valine. It is somewhat remarkable that this relatively small change of leucine to valine make such a significant difference in protein stability. The importance of the role for this leucine may explain its complete conservation in vertebrates. Consistent with LynΔN being an N-End rule substrate is the proteasome, UBR1 and UBR2 dependent degradation.

The degradation of LynΔN by the N-end rule pathway is an additional example of how the N-End rule pathway can function in a pro-apoptotic function. This was demonstrated by how the valine-LynΔN stabilizing mutant provides significantly higher imatinib resistance to K562 cells. A previous example of N-End rule dependent cell death has been previously described with macrophages treated with anthrax lethal toxin[26]. In conjunction with previous reports demonstrating anti-apoptotic functions of the N-End rule in other pathways[21, 23], it is becoming apparent that the role of the N-end Rule pathway cannot be specifically labeled as a pro- or anti-apoptotic pathway. The commonality of the reported examples of proteins being targeted for degradation is that the N-End rule counters the proteolytic activation of these proteins.

## MATERIALS & METHODS

### Generation of ubiquitin fusion cleaved Lyn expression vector

To express the cleaved Lyn fragment we cloned the cleaved Lyn (isoform 1) as a fusion between an N-terminal ubiquitin and C-terminal triple FLAG tag (3x FLAG) that had been previously cloned in a pcDNA 3.1 hygro plasmid[24]. A cDNA clone (clone ID: MHS6278-211689273, Open Biosystems) was used for cloning the Lyn sequence, and the sequence used corresponds to amino acids 19 to 512. This sequence of Lyn corresponds to the sequence from the known caspase cleavage site[13] to the C-terminal end of the protein.

### Site directed mutagenesis

Mutagenesis of the codon for the leucine corresponding to the N-termini of the cleaved Lyn protein were performed by site directed mutagenesis to change the codon to arginine (CGG), valine (GUG) and methionine (AUG). Similarly, the kinase dead form of cleaved Lyn was obtained through mutating the lysine residue at position 275 in the putative ATP-binding site to arginine (CGG) by site directed mutagenesis.

### Cell culture

HEK293T cells were obtained from the ATCC and K562 cells were obtained from Dr. Chris Bleakly (University of Alberta, Canada). The HEK293T cells were cultured in DMEM supplemented with 10 % fetal bovine serum (FBS). K562 cells were cultured in RPMI1640 with 10% FBS.

### Cell transfection

HEK293T were transfected using the Calcium phosphate-based method as previously described[31]. K562 cells were transfected using electroporation (Neon transfection system) according to manufacturer's procedures (Pulse voltage 1450 v, Pulse width (ms) 10 and 3 pulses).

### Reagents and antibodies

DAPI was purchased from Sigma (Saint Louis, MO, USA). PARP antibody (cat#: 9542) was purchased from cell signalling technology (Beverly, MA, USA). Mouse anti-FLAG® M2 antibody (cat#: F1804) was purchased from Sigma. Rabbit anti-β-actin (I-19, cat#: sc-1616-R), anti-UBR1 (cat#: sc-100626) and anti-UBR2 (cat#: sc-135594) were purchased from Santa Cruz Biotechnology. Secondary antibodies for Western blot analysis (goat anti-mouse and goat anti-rabbit) coupled to IRDyes® were purchased from LI-COR. The Alexa Fluor 488-labeled secondary goat anti-mouse antibody for immunostaining was purchased from Invitrogen. Rabbit Anti-GFP antibody was a gift from Luc Berthiaume (Department of Cell Biology, University of Alberta).

### Western blotting

After SDS-PAGE (on 5 or 10% gels), proteins were transferred onto nitrocellulose membranes (LI-COR Biosciences). The membranes were blocked with 2.5% fish skin gelatin blocking buffer (0.5% of Cold Water Fish Skin Gelatin (Sigma) in 1× phosphate buffered saline - pH 7.4 with 0.1% Triton X-100) and probed with primary and secondary antibodies and imaged with an Odyssey Infrared Imaging System using the manufacturer's recommended procedures (LI-COR).

### CHX-chase assays and Western blot analysis

24 hrs after transfection, 5×10^5^ cells were treated with 100μg/ml of cycloheximide for the indicated amounts of time. Cells were harvested and then lysed in 150 μl of lysis buffer (50 mm Tris, pH 6.8, 8% glycerol (v/v), 0.016% SDS (w/v), 0.125% β-mercaptoethanol (v/v), 0.125% bromphenol blue (w/v), 1 mm PMSF, and 1 μg/ml of leupeptin). The samples were sonicated for 10 seconds with an amplitude of 30% (1 watt), then resolved by SDS-PAGE on 10% gels along with Precision Plus All Blue protein pre-stained standards (Bio-Rad) as molecular weight markers. After SDS-PAGE, proteins were transferred onto nitrocellulose membranes (LI-COR Biosciences). The membranes were blocked with fish skin gelatin blocking buffer (2.5% of Cold Water Fish Skin Gelatin in PBS and 0.1% Triton X-100) and probed with primary and secondary antibodies and imaged with an Odyssey® Infrared Imaging System using the manufacturer's recommended procedures (LI-COR).

### Fluorescence immunostaining and imaging

Transiently transfected HEK 293T were seeded on coverslips in 12-well plates (2.5 × 10^3^ cells/well) and grown for 48 hrs. Cells were then washed with PBS+ (PBS containing 1 mM MgCl_2_ and CaCl_2_) and fixed in 4% paraformaldehyde for 15 min at 22 °C. The cells were then permeabilized with 0.1% Triton X-100 for 2 min at 22 °C and then washed three times with PBS+. The cells were then blocked with 4% normal donkey serum at 22 °C for 1 hr and washed again with PBS+ prior to incubation with the 1° Antibody (1 in 150 dilution in 4% normal donkey serum) for 1 h at 22 °C. After a washing step (three washes with PBS+) the cells were then incubated with the 2° Antibody (1:200 dilution in 4% normal donkey serum) for 60 min at 22 °C in the dark. After washing, the cells were stained with DAPI nuclear counterstain (1:1000 dilution). After a single wash step the coverslips were mounted on slides using Dako Fluorescence mounting medium and allowed to set overnight prior to imaging. Fluorescence images were obtained with an Axiocam on an Axio Observer microscope (Carl Zeiss, Jena, Germany) using a ×100 Plan Aprochromat Lens.

### UBR1 and UBR2 shRNAs

Four UBR1 unique 29mer shRNA constructs in GFP-V-RS vectors (cat#: TG300681) were purchased from Origene. Four unique UBR2 29mer shRNA constructs in RFP-C-RS vectors (cat#: TF300680) were also purchased from Origene.

### Cell viability assay

Cell counting and the trypan blue exclusion test were performed with a TC20™ Automated Cell Counter (BioRad).

### Flow cytometry

Annexin V-FITC Apoptosis detection Kit (eBioscience) was used for apoptosis analysis by flow cytometry. Cells (5 × 10^5^ cells) were incubated for 10 minutes in 500 μl 1× binding buffer, 5 μl of Annexin V-FITC and 10ul of PI (20 μg /ml) prior to flow cytometry analysis on a LSR-Fortessa Instrument. Ten thousands events are acquired for statistical analysis.
